# High glucose levels affect retinal patterning during zebrafish embryogenesis

**DOI:** 10.1038/s41598-019-41009-3

**Published:** 2019-03-11

**Authors:** Amitoj Singh, Hozana Andrade Castillo, Julie Brown, Jan Kaslin, Karen M. Dwyer, Yann Gibert

**Affiliations:** 1 Deakin University, School of Medicine, Faculty of Health, 75 Pigdons Road, Waurn Ponds, Geelong, VIC 3216 Australia; 2 Monash University, Australian Regenerative Medicine Institute, 23 Innovation Walk, Clayton, VIC 3800 Australia; 3 Brazilian Biosciences National Laboratory, Brazilian Centre for Research in Energy and Materials, Campinas, Brazil

## Abstract

Maternal hyperglycaemia has a profound impact on the developing foetus and increases the risk of developing abnormalities like obesity, impaired glucose tolerance and insulin secretory defects in the post-natal life. Increased levels of glucose in the blood stream due to diabetes causes visual disorders like retinopathy. However, the impact of maternal hyperglycaemia due to pre-existing or gestational diabetes on the developing foetal retina is unknown. The aim of this work was to study the effect of hyperglycaemia on the developing retina using zebrafish as a vertebrate model. Wild-type and transgenic zebrafish embryos were exposed to 0, 4 and 5% D-Glucose in a pulsatile manner to mimic the fluctuations in glycaemia experienced by the developing foetus in pregnant women with diabetes. The zebrafish embryos displayed numerous ocular defects associated with altered retinal cell layer thickness, increased presence of macrophages, and decreased number of Müeller glial and retinal ganglion cells following high-glucose exposure. We have developed a model of gestational hyperglycaemia using the zebrafish embryo to study the effect of hyperglycaemia on the developing embryonic retina. The data suggests that glucose exposure is detrimental to the development of embryonic retina and the legacy of this exposure may extend into adulthood. These data suggest merit in retinal assessment in infants born to mothers with pre-existing and gestational diabetes both in early and adult life.

## Introduction

It is well established that chronic hyperglycaemia induces changes in the retina and vision^1^. Hyperglycaemia incites pro-inflammatory reactions leading to the breakdown of the blood-retinal barrier and activation of the normally quiescent microglial cells resulting in the production of reactive oxygen species, culminating in neuronal cell death and vision loss^2–4^. In addition, neovascularization of the retinal blood vessels occurs, leads to the abnormal proliferation of leaky and fragile blood vessels on the surface of the retina that haemorrhage profusely. The accumulation of blood in the vitreous chamber and retinal detachment may culminate in blindness^5^. Indeed, diabetes is the leading cause of blindness in the western world^6^.

Glucose, produced by maternal metabolism from carbohydrates in the diet, is the primary substrate for intrauterine growth^7,8^. Hyperglycaemia during pregnancy due to pre-existing or gestational diabetes mellitus leads to increased nutrient transfer to the foetus, resulting in increased birth weight^9,10^ and impaired glucose tolerance, insulin secretory defects and obesity in later life^9,11–13^. Knowledge regarding the impact of hyperglycaemia on the developing retina is lacking, which we sought to address in this study.

Zebrafish is becoming a valuable tool in the study of various physiological and pathological conditions like diabetes leading to the development of cardiovascular diseases, impairment of brain function and visual disorders in vertebrates^14–16^. Several factors like the ability to mimic persistent hyperglycaemia as experienced by diabetic individuals^17,18^ make zebrafish amenable to the study of diabetes induced microvascular complications like retinopathy^19–21^. In addition, the ability of the zebrafish retina for regeneration also make zebrafish an attractive vertebrate animal model^22^.

We utilised zebrafish as a vertebrate animal model to study hyperglycaemia induced changes in the retina as the zebrafish eye shares structural similarities with the human eye such as presence of the lens, retinal ganglion layer (RGL), inner plexiform layer (IPL), the inner nuclear layer (INL), the outer plexiform layer (OPL) and the outer nuclear layer (ONL). In addition, transgenic lines in zebrafish allow live fluorescent imaging of retina specific cells, making them an attractive model to study various ocular disorders. We have addressed two key knowledge gaps in this study. First, the effect of hyperglycaemia on retinal formation during embryonic development and secondly, the effect of hyperglycaemia during embryogenesis on the adult retina.

## Results

### Exposure to high concentrations of glucose induces hyperglycaemia in zebrafish embryos

**Figure 1:**
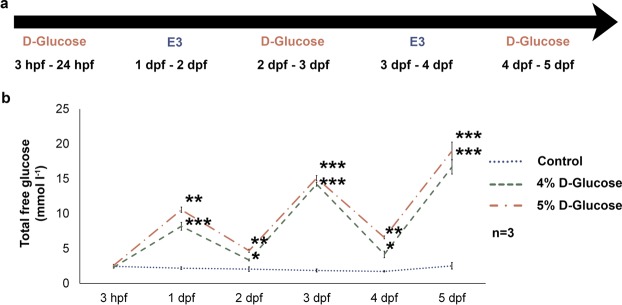
Treatment timeline and measurement of total free glucose levels in zebrafish embryos. (**a**) WT zebrafish embryos were exposed to 4 and 5% D-Glucose in embryonic media (E3) from 3 hpf until 5 dpf. Control zebrafish remained in the vehicle (E3) only under identical conditions. The environment of the embryos was alternated every 24 h between the vehicle and freshly prepared 4 and 5% D-Glucose solution. **(b)** Total Free Glucose levels measured at 3 hpf, 1, 2, 3, 4 and 5 dpf exhibit a dose-dependent fluctuation of the total free glucose levels from 3 hpf until 5 dpf. Error bars indicate mean ± s.e.m. Statistical differences were computed using two-tailed student’s t-test and are indicated as *p < 0.05, **p < 0.005, ***p < 0.0005, (n = 3).

Blood glucose levels are normally maintained within a narrow range^23^. This precise physiological mechanism is compromised in women with pre-existing or gestational diabetes^24^. To simulate changing blood glucose levels, we exposed WT zebrafish embryos to 4 and 5% D-Glucose in a pulsatile manner from 3 hpf until 5 dpf (Fig. 1a). Following treatment from 3 hpf until 5 dpf, control embryos exhibited a mortality rate of 4%, while the 4 and 5% D-Glucose treated zebrafish showed a mortality rate of 28.4 and 37.5%, respectively. The average total free glucose level fluctuated in line with exposure to 4 and 5% D-Glucose and E3 media, respectively, reaching 16.7 ± 1.01 mmol l^−1^ (mean ± s.e.m.) for the 4% D-Glucose treated group, and 19 ± 1.26 mmol l^−1^ (mean ± s.e.m.) for embryos treated with 5% D-Glucose at 5 dpf (Fig. 1b). Total free glucose level was stable (2.14 ± 0.13 mmol l^−1^) in control WT embryos (Fig. 1b).

We chose to proceed with only 4% D-Glucose exposure for some experiments as the retina is reported to have a reduced ability to change glucose transport rates when faced with excessive extracellular glucose concentrations^25^.

### High glucose exposure in zebrafish embryos alters the morphology of the eye and thickness of the retinal cell layers

**Figure 2:**
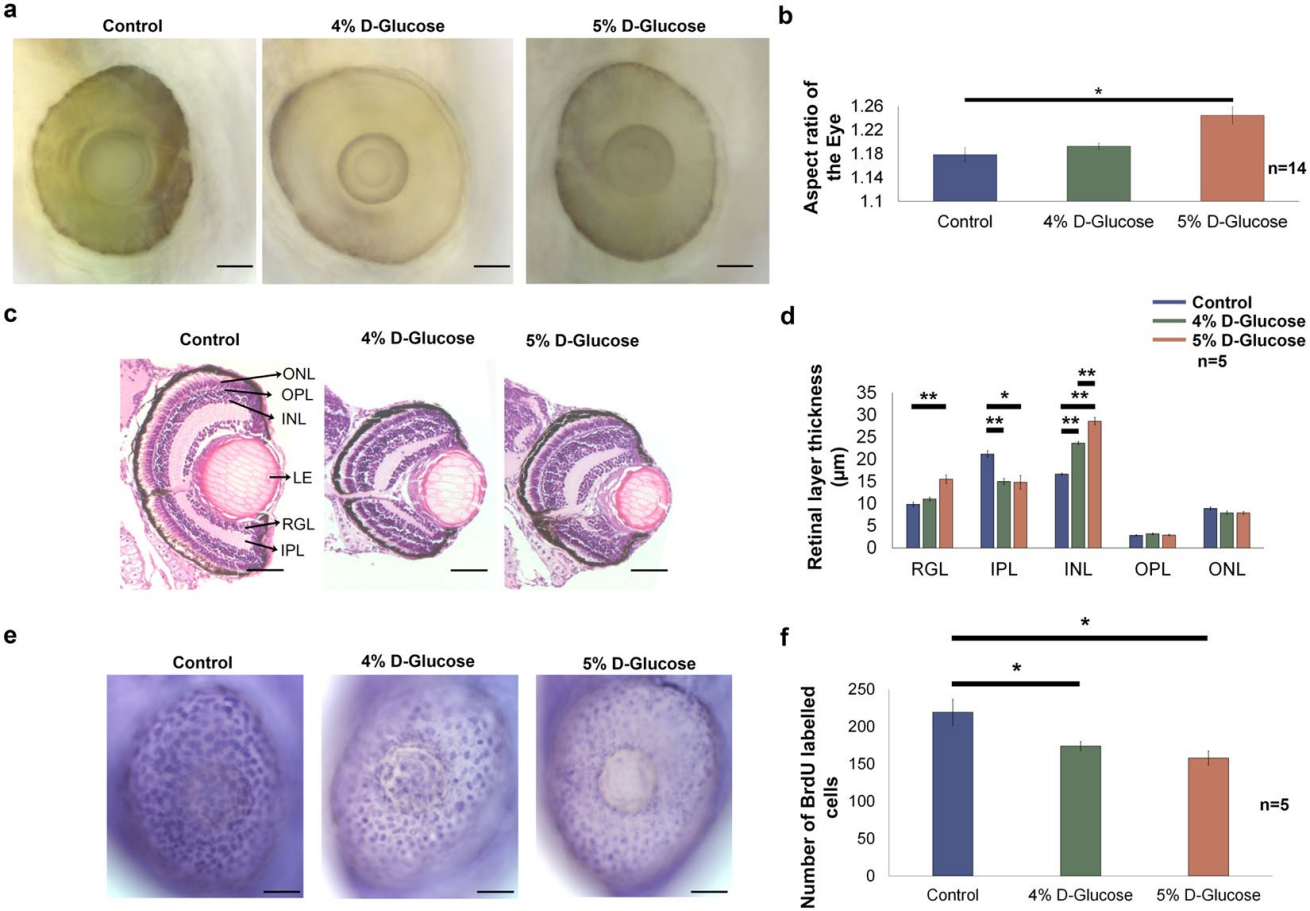
Hyperglycaemia induced changes acquired by the embryonic zebrafish eye. **(a)** Lateral view of the embryonic zebrafish eye following pulsatile 4 and 5% D-Glucose treatment from 3 hpf until 5 dpf. Control zebrafish were maintained in the vehicle only from 3 hpf until 5 dpf under identical conditions. Scale bar, 50 µm. **(b)** The aspect ratio of the embryonic zebrafish eye remained unchanged following pulsatile 4% D-Glucose exposure. The aspect ratio of the embryonic zebrafish eye significantly increased following pulsatile 5% D-Glucose treatment from 3 hpf until 5 dpf as compared to controls reflecting an alteration in the morphology of the eye. Error bars indicate mean ± s.e.m.; Statistical differences were computed using two-tailed student’s t-test and are indicated as *p = 0.001, (n = 14). **(c)** Hematoxylin and Eosin staining depicting representative images of the cross-sections of the embryonic zebrafish eye. WT embryos exposed to 4 and 5% D-Glucose from 3 hpf until 5 dpf in a pulsatile manner exhibited an alteration in the thickness of retinal layers at 5 dpf. Controls remained in identical conditions in the vehicle (E3) only. Scale bar, 50 µm. **(d)** Statistical analysis demonstrated that WT embryos exposed to 4% D-Glucose from 3 hpf until 5 dpf in a pulsatile manner developed a significant thickness of the INL and a decrease in the thickness of IPL as compared to the control embryos at 5 dpf. WT embryos exposed to 5% D-Glucose from 3 hpf until 5 dpf in a pulsatile manner showed the significantly decreased thickness of IPL, and an increase in the thickness of the RGL and INL, while other retinal layers remained unaffected. Error bars indicate mean ± s.e.m.; Statistical differences were computed using two-tailed student’s t-test and are indicated as *p = 0.001, **p < 0.0005; Results were obtained from representative sections from 5 zebrafish embryos, n = 5. **(e)** Lateral view of the BrdU labelled embryonic zebrafish eye at 5 dpf following pulsatile 4 and 5% D-Glucose exposure from 3 hpf until 5 dpf. Controls remained in identical conditions in the vehicle (E3) only. Arrows represent BrdU labelled retinal cells. Scale bar, 50 µm. **(f)** Statistical analysis revealed a significant decrease in BrdU labelled retinal cells following 4 and 5% D-Glucose exposure compared to untreated controls at 5 dpf. Error bars indicate mean ± s.e.m. Statistical differences were computed using two-tailed student’s t-test and are indicated as *p < 0.05. Results were obtained from representative images from 5 zebrafish embryos (n = 5). ONL, outer nuclear layer; OPL, outer plexiform layer; INL, inner nuclear layer; LE, lens; RGL, retinal ganglion layer; IPL, inner plexiform layer.

WT embryos exposed to 4% and 5% D-Glucose from 3 hpf until 5 dpf in a pulsatile manner exhibited a significantly increased aspect ratio (ratio of the maximum and minimum diameter of the eye) compared to controls, suggesting a general change in the ‘elliptical’ morphology of the eye (Fig. 2a,b). Following pulsatile D-Glucose exposure, WT embryos showed a significantly reduced thickness of IPL (29.4% for 4% and 30.14 for 5%) at 5 dpf and a significant increase in the thickness of the INL (42.3% for 4% and 71.9% for 5%) while the OPL and ONL remained unaffected (Fig. 2c,d). In addition, only embryos exposed to pulsatile 5% D-Glucose treatment exhibited an increase in the thickness of RGL (57.7%) (Fig. 2c,d).

To identify the developmental stage when the retinal cell layers are most susceptible to high-glucose induced histological changes, WT embryos were exposed to 4% D-Glucose from 24 until 48 hpf, 72 until 96 hpf, and 96 until 120 hpf. These time points were chosen as from 24–48 hpf the retina has not differentiated into well-defined cell layers^26^; 72–96 hpf coincides with the development of fully differentiated neurons^27^, and by 96–120 hpf well-defined lamina and clearly defined retinal cell layers are evident^28^.

**Figure 3:**
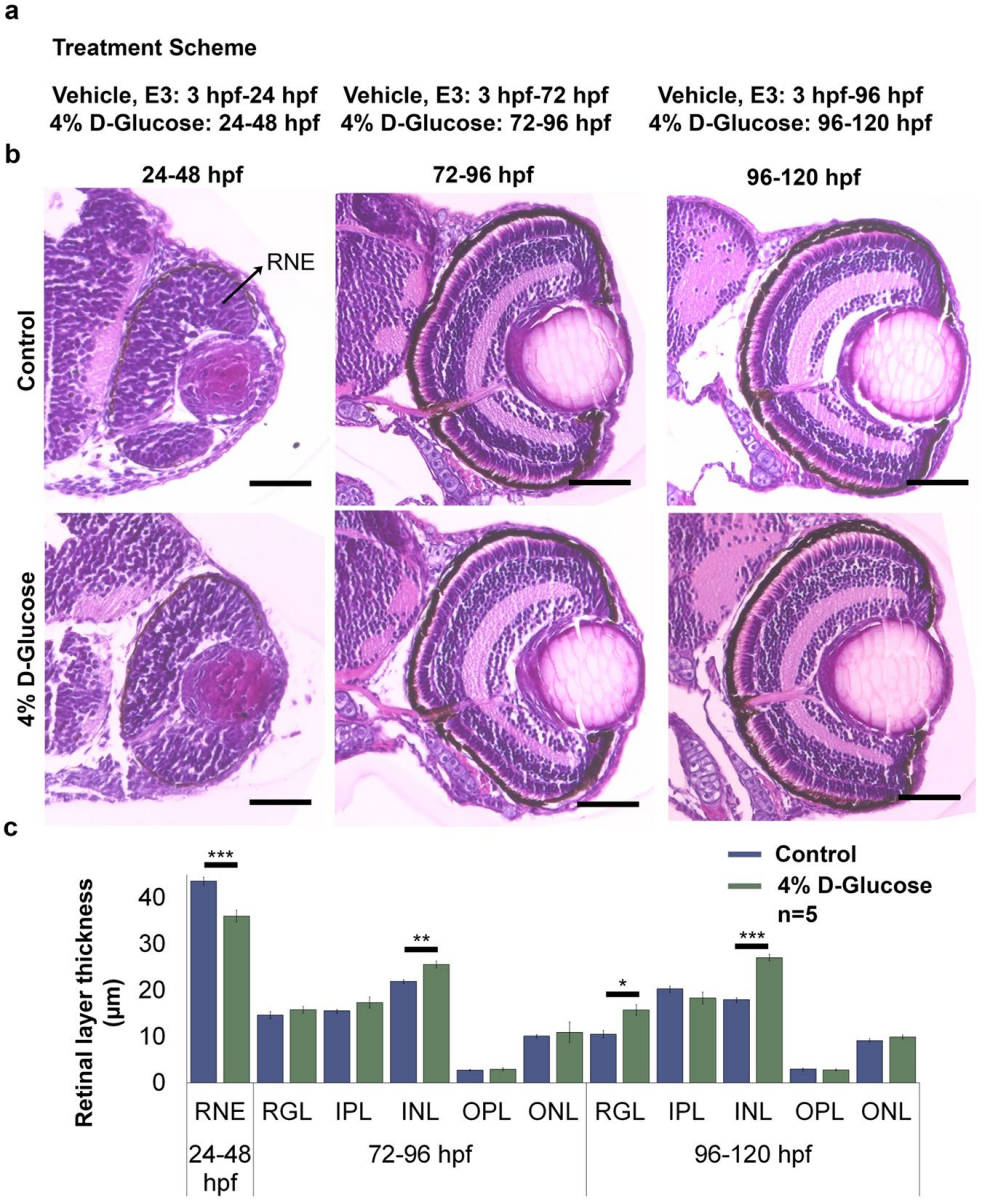
Changes in the retinal layer thickness following 4% D-Glucose exposure at various time points. **(a)** Treatment scheme. WT embryos were maintained in vehicle until 24, 72 and 96 hpf, followed by a 24 h treatment with 4% D-Glucose until 48, 96 and 120 hpf, respectively. Control embryos were maintained in vehicle until 48, 96 and 120 hpf. **(b)** Representative images of Hematoxylin and Eosin staining obtained from sections depicting cross-sections of the control and 4% D-Glucose treated embryonic zebrafish eye at 48, 96 and 120 hpf. WT embryos exposed to a 24 h 4% D-Glucose treatment at 24, 72 and 96 hpf exhibited an alteration in the retinal layer thickness at 48, 96 and 120 hpf, respectively. Scale bar, 50 µm **(c)** Statistical analysis demonstrated that WT embryos exposed to 4% D-Glucose at 24 hpf exhibited a significant decrease in the thickness of the RNE at 48 hpf compared to the controls. WT embryos exposed to 4% D-Glucose at 72 and 96 hpf for 24 h showed a significant increase in the thickness of the INL at 96 hpf, and the INL and RGL at 120 hpf, respectively. Controls remained in similar conditions in the vehicle (E3) only. Error bars indicate mean ± s.e.m.; Statistical differences were computed using two-tailed student’s t-test and are indicated as *p < 0.005, **p = 0.0005, ***p < 0.0005; Results were obtained from representative sections from 5 zebrafish embryos, n = 5. RNE, retinal neural epithelium; ONL, outer nuclear layer; OPL, outer plexiform layer; INL, inner nuclear layer; LE, lens; RGL, retinal ganglion layer; IPL, inner plexiform layer.

Exposure of WT embryos to 4% D-Glucose from 24 until 48 hpf significantly decreased the thickness of the retinal neural epithelium (RNE) by 17.3% as compared to untreated controls (Fig. 3a,b). Exposure between 72 and 96 hpf did not affect the RGL, IPL, OPL and ONL, however, INL thickness was significantly increased by approximately 16% as compared to age-matched controls (Fig. 3a,b). Exposure between 96 and 120 hpf significantly increased the thickness of only the RGL and INL by 49% and 50%, respectively as compared to controls (Fig. 3a,b). These data indicate that high glucose exposure has a differential effect on the retinal cell layers according to the time of exposure.

### Retinal cell proliferation is reduced following high glucose exposure

Analysis of BrdU labelled retinal cells following 4 and 5% D-Glucose exposure from 3 hpf until 5 dpf in a pulsatile manner revealed a significant decrease in the number of BrdU labelled cells (20.6% for 4% and 27.9% for 5%) compared to untreated controls at 5 dpf (Fig. 2e,f). This suggests that the early embryonic retinal cell population is sensitive to glycaemic changes and cell proliferation is reduced following hyperglycaemia.

### High glucose exposure reduces retinal ganglion and Müeller glial cell numbers

**Figure 4:**
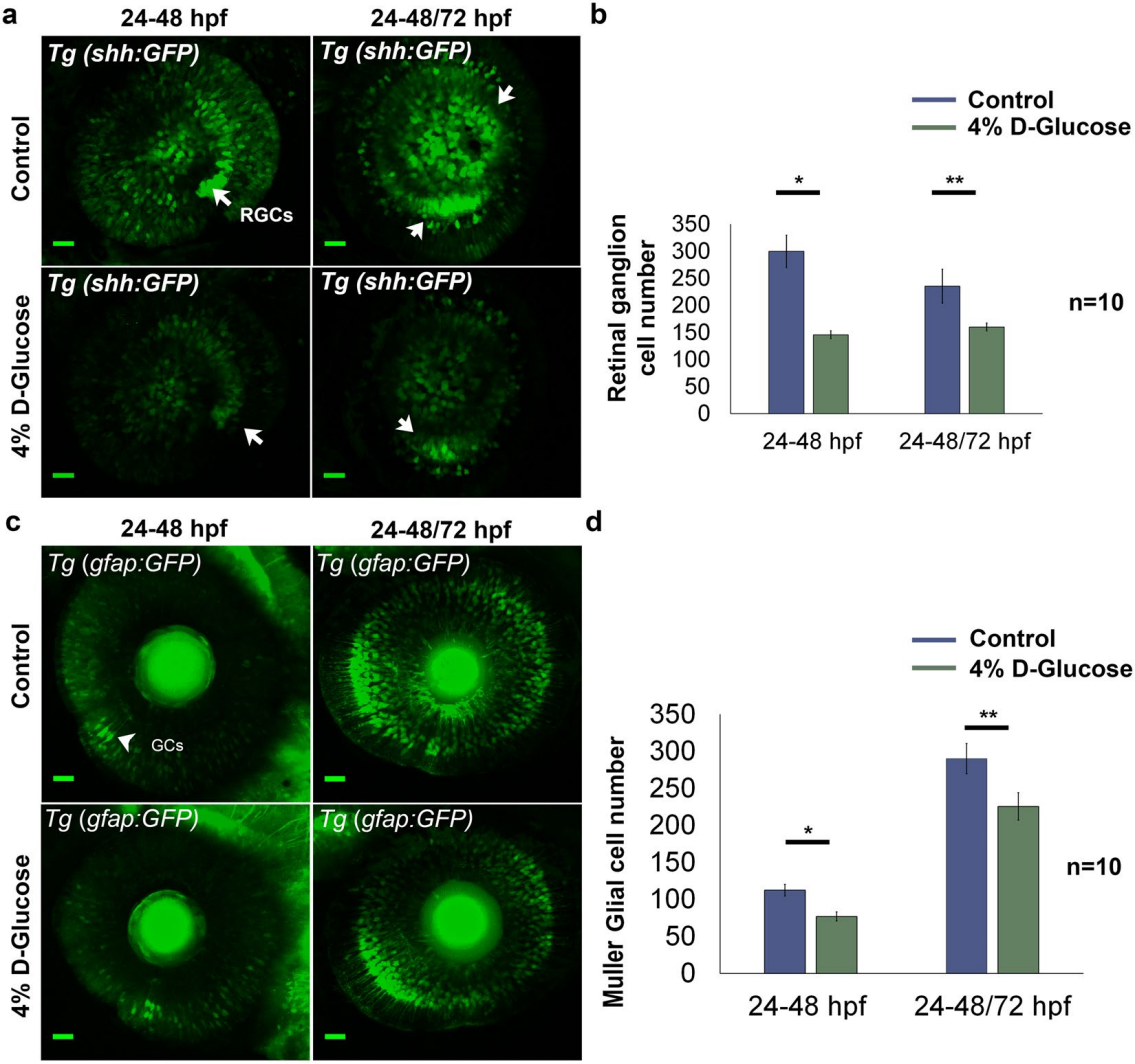
Changes in retinal cell population following 4% D-Glucose exposure. (**a)** Reduced number of retinal ganglion cells (RGCs) (indicated by white arrows) following 4% D-Glucose exposure. *Tg (shh:GFP)* embryos were exposed to 4% D-Glucose at 24 hpf, and imaged at 48 hpf. *Tg (shh:GFP)* embryos were allowed to recover in the vehicle (E3) between 48 and 72 hpf, following 4% D-Glucose exposure between 24–48 hpf, and imaged at 72 hpf. **(b)** *Tg (shh:GFP)* exhibited a significant decrease in the number of RGCs at 48 hpf. Following, recovery in the vehicle between 48 and 72 hpf, *Tg (shh:GFP)* imaged at 72 hpf failed to recover and exhibited significantly reduced RGCs. Error bars indicate mean ± s.e.m.; Statistical differences were computed using two-tailed student’s t-test and are indicated as *p = 0.006, **p < 0.0005; (n = 10). Scale bar, 20 µm. **(c)** Reduced number of Müeller glial cells (GCs) (indicated by white arrowheads) following 4% D-Glucose exposure. *Tg (gfap:GFP)* embryos were exposed to 4% D-Glucose at 24 hpf, and imaged at 48 hpf. *Tg (gfap:GFP)* embryos were allowed to recover in vehicle only between 48 and 72 hpf, following 4% D-Glucose exposure between 24 and 48 hpf, and imaged at 72 hpf. **(d)** *Tg (gfap:GFP)* exhibited a significant decrease in the number of GCs at 48 hpf. Following, recovery in the vehicle between 48 and 72 hpf, *Tg (gfap:GFP)* imaged at 72 hpf failed to recover and exhibited significantly reduced GCs. Error bars indicate mean ± s.e.m.; Statistical differences were computed using two-tailed student’s t-test and are indicated as *p = 0.01, **p < 0.03; (n = 10). Scale bar, 20 µm. RGCs, retinal ganglion cells; GCs, Müeller glial cells.

*Tg (shh:GFP)* embryos (with GFP labelled retinal ganglion cells, RGCs) were exposed to 4% D-Glucose from 24 until 48 hpf and imaged at 48 hpf using confocal microscopy. The number of RGCs in the *Tg (shh:GFP)* retina significantly decreased by 51.3% (n = 10, p < 0.05) as compared to controls (Fig. 4a,b). Another group of *Tg (shh:GFP)* embryos was transferred to vehicle for 24 h following 4% D-Glucose exposure (from 24 until 48 hpf) and imaged at 72 hpf. The number of RGCs remained significantly decreased by 31.9% compared to controls at 72 hpf (n = 10, p < 0.0005) after the 24 h recovery period (Fig. 4a,b).

Exposure of *Tg (gfap:GFP)* embryos (with GFP labelled Müeller glial cells) to 4% D-Glucose from 24 until 48 hpf significantly reduced the number of Müeller glial cells in the retina by 37.1% compared to controls (n = 10, p < 0.05) (Fig. 4c,d). The reduction in Müeller glial cell number following 4% D-Glucose exposure (between 24 and 48 hpf) was sustained (22.2% of the controls at 72 hpf) following a further 24 h recovery in E3 media alone (n = 10, p < 0.005) (Fig. 4c,d). These data suggest that the decrease in the number of RGCs and Müeller glial cell induced by hyperglycaemia are not rescued post glucose exposure.

### Hyperglycaemia during development induces microvascular changes and retinal blood vessel leakage

**Figure 5:**
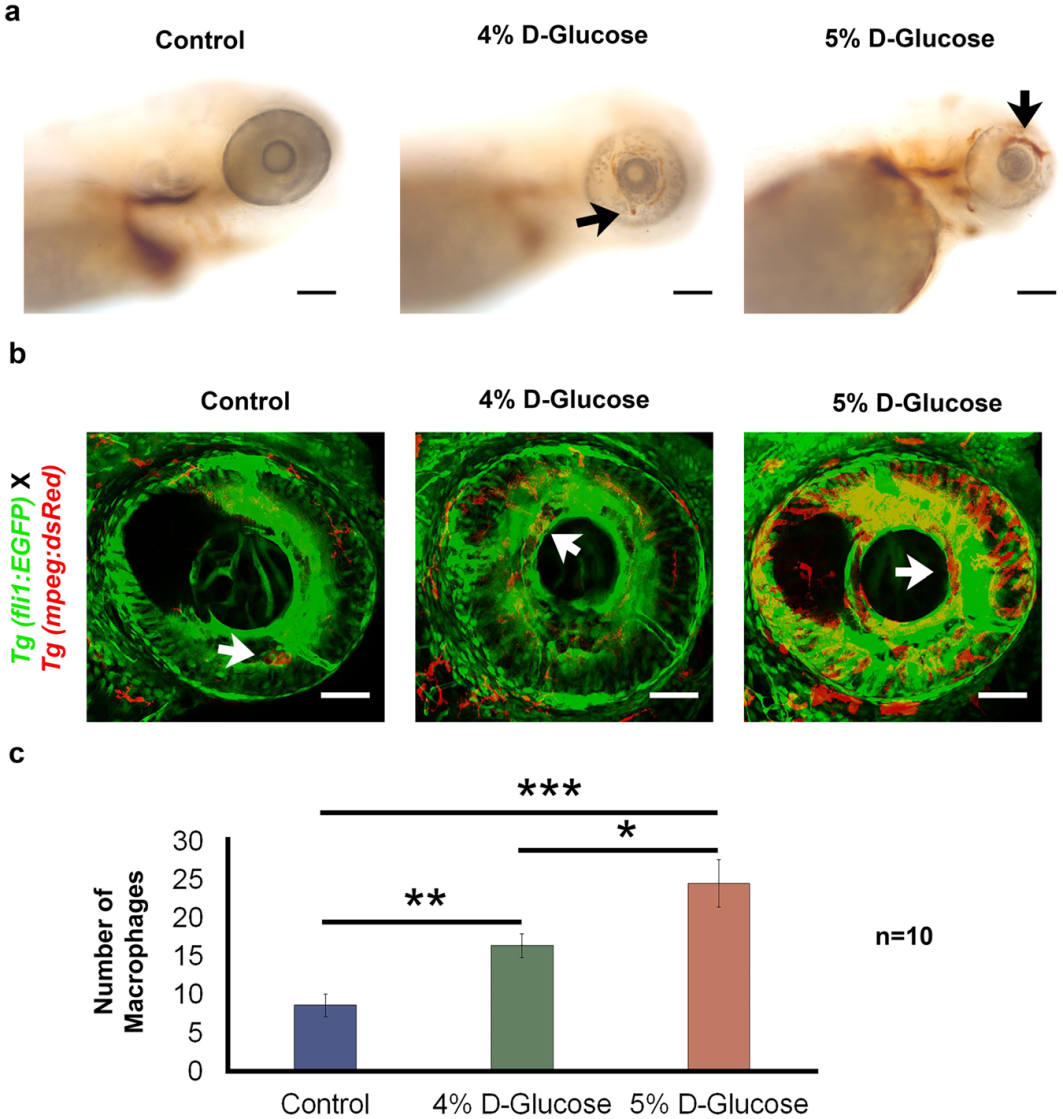
Increased haemoglobin presence and retinal blood vessel leakage following pulsatile high-glucose exposure from 3 hpf until 5 dpf. (**a**) The increased presence of haemoglobin in the retina (indicated with black arrows) was detected in WT embryos exposed to 4 and 5% D-Glucose in a pulsatile manner from 3 hpf until 5 dpf following O-dianisidine staining indicating increased accumulation of hemoglobin in the retinal blood vessels, which is indicative of leaky retinal blood vessels. Control WT embryos were maintained in the vehicle (E3) only from 3 hpf until 5 dpf under standard conditions. (n = 5). Scale bar, 50 µm. (**b**) Retinal blood vessel leakage following pulsatile D-Glucose exposure. *Tg (fli1:EGFP) X Tg (mpeg:DsRed)* embryos were exposed to a fluctuating 4 and 5% D-Glucose treatment from 3 hpf until 5 dpf. Scale bar, 50 µm. (**c**) Statistical analysis of *Tg (fli1:EGFP) X Tg (mpeg:DsRed)* exhibited an increase in the number of macrophages (indicated by white arrows) in the retina at 5 dpf, indicating blood vessel leakage. Error bars indicate mean ± s.e.m.; Statistical differences were computed using two-tailed student’s t-test and are indicated as *p = 0.03, **p = 0.007, ***p < 0.001; (n = 10).

O-dianisidine staining was used to detect haemoglobin in the embryonic retina. WT embryos exposed to fluctuating 4 and 5% D-Glucose treatment from 3 hpf until 5 dpf showed an increased accumulation of haemoglobin in the retina indicative of microvascular changes in the choroid and hyaloid retinal blood vessels (Fig. 5a). These data are in contrast to controls, which showed minimal accumulation of the haemoglobin at 5 dpf (n = 5) (Fig. 5a).

Double transgenic embryos obtained from a cross of adult *Tg (fli1:EGFP) X Tg (mpeg:DsRed)* zebrafish (double transgenic zebrafish embryos with EGFP labelled blood vessels and DsRed labelled macrophages), and exposed to pulsatile 4% D-Glucose treatment from 3 hpf until 5 dpf, accumulated 89.8% more macrophages in the retina as compared to controls during the same time period (n = 10, p < 0.005) (Fig. 5b,c). *Tg (fli1:EGFP) X Tg (mpeg:DsRed)* embryos exposed to 5% D-Glucose within the same time period exhibited a dose-dependent increase in the accumulation of macrophages; approximately 49% more as compared to 4% D-Glucose (n = 10, p < 0.05), and approximately 183% as compared to controls within the same time period (n = 10, p < 0.001) (Fig. 5b,c). Together these data indicate that hyperglycaemia promotes the accumulation of macrophages and choroid and hyaloid retinal blood vessel fragility and haemorrhage in the embryonic retina.

### High glucose level during embryonic development increases the number of hyaloid blood vessel sprouts in adults

**Figure 6:**
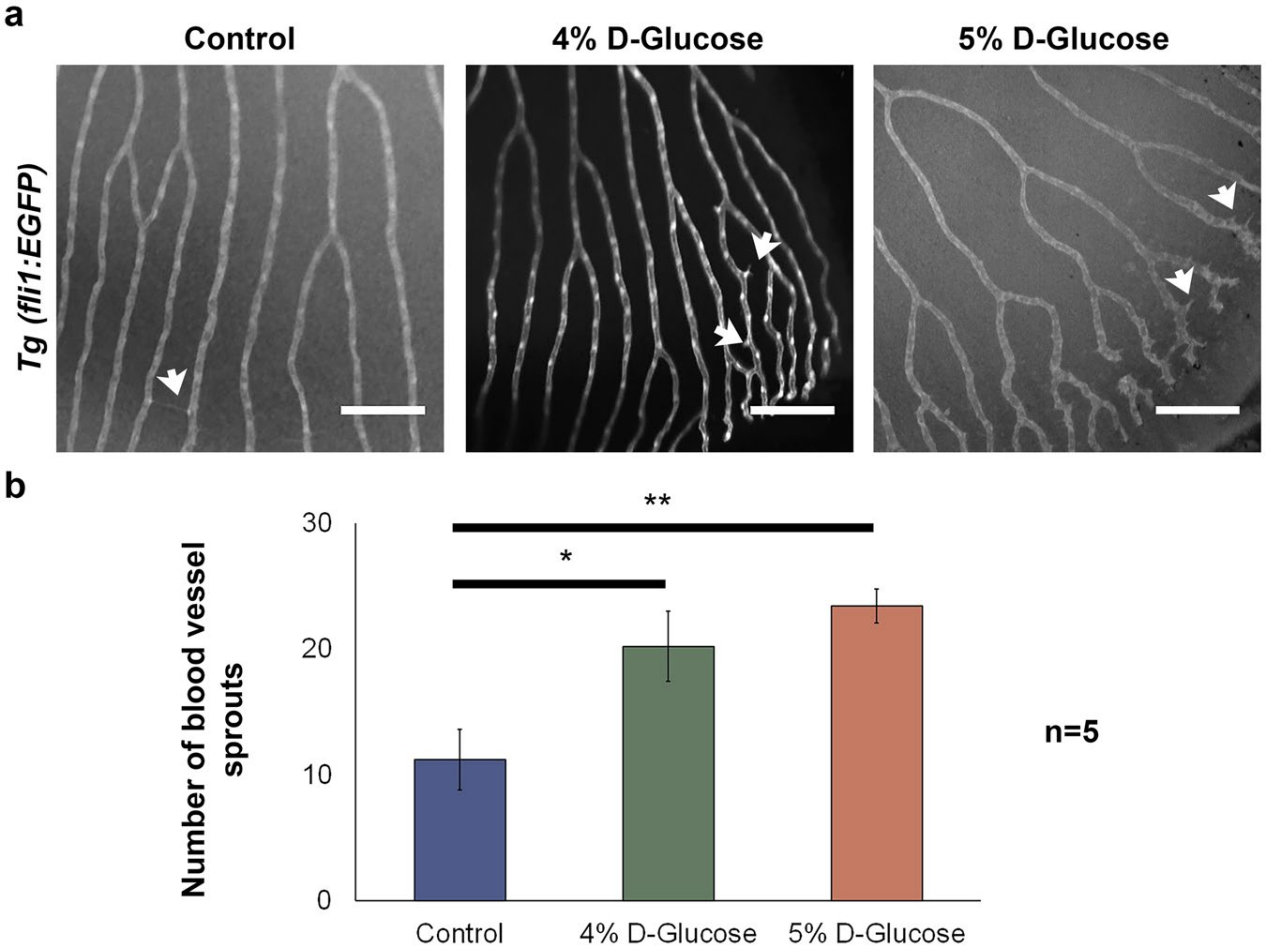
Long-term implications of high-glucose exposure during embryonic stages in the adult zebrafish retina. (**a)** Retinal vasculature of *Tg (fli1:EGFP)* zebrafish adults (100 dpf) exposed to vehicle, 4 and 5% D-Glucose from 3 hpf until 5 dpf in a pulsatile manner. 4 and 5% D-Glucose treated group exhibited an increase in the number of hyaloid blood vessel sprouts (indicated by white arrows). **(b)** Statistical analysis of the number of blood vessel demonstrated a significant increase in the number of hyaloid blood vessel sprouts. Error bars indicate mean ± s.e.m.; Statistical differences were computed using two-tailed student’s t-test and are indicated as *p = 0.02, **p = 0.003; (n = 5). Scale bar, 100 µm. Images inverted to grayscale.

In order to investigate the long-term effects of embryonic high-glucose exposure on the adult retina, *Tg (fli1:EGFP)* embryos were exposed to vehicle and, 4 and 5% D-Glucose from 3 hpf until 5 dpf using the pulsatile method and raised to 100 dpf under standard conditions^29^. We found a significant increase in the number of hyaloid retinal blood vessel sprouts (20.2 ± 2.7 for the 4% and 23.4 ± 1.3 for the 5% D-Glucose group, mean ± s.e.m.) compared to the control group (11.2 ± 2.4, mean ± s.e.m.) (Fig. 6a,b) (Refer Fig. S1 for biological replicates). These data suggest that hyperglycaemia during the embryonic development of the retina has implications for the adult retina.

## Discussion

Hyperglycaemia associated with established diabetes causes a variety of neurogenic disorders in the human body. These include impairment of neurogenic processes and neural plasticity^30,31^, and impairment of cognitive functions such as memory^32^. The major complication of diabetes arising due to hyperglycaemia are progressive visual disorders leading to partial or total vision loss in affected individuals^33,34^. Hyperglycaemia induces a variety of detrimental changes in the retina such as micro-aneurysms, haemorrhages, retinal oedema^34^, photoreceptor loss and dysfunction^35,36^. Glucose concentrations in pregnant women with pre-existing or gestational diabetes have been reported to be up to three times that of women without diabetes at a similar gestation^37^. *In-utero*, the glucose levels experienced by the human foetus depends on the nutritional status and metabolic profile of the mother. Although maternal hyperglycaemia has profound effects on the offspring both immediately and in adulthood^38^, the impact on the retina has not yet been clearly identified. In the absence of reliable animal models that correctly recapitulate the *in-utero* exposure of glucose in human beings, zebrafish embryos provide a means of direct glucose exposure and the analysis of retinal changes.

In this study, we used D-Glucose as an exogenous agent to simulate hyperglycaemic foetal microenvironment in zebrafish embryos within the range comparable to existing animal models^39–41^ and to mimic the wide fluctuations in glycaemia experienced by humans^42^. Depending on the severity, glucose levels in diabetic patients ranges between 5.6 and >33.3 mmol l^−1^ ^43–45^. Zebrafish embryos exhibited total free glucose levels between 8.2 ± 0.55 mmol l^−1^ to 19 ± 1.26 mmol l^−1^ (Fig. 1b) depending on the concentration of glucose (4 or 5%) that the embryos were exposed to, making our free glucose concentration right in the range of what has been reported in humans^43,44^. Therefore, the zebrafish is a highly appropriate model to study hyperglycaemia related visual disorders as: i, zebrafish embryos exhibited a fluctuating hyperglycaemia, well within the range reported in diabetic patients and ii, because of its external mode of development, the zebrafish embryo allows direct exposure of glucose, bypassing any limitations due to the placental-foetal barrier present in mammals.

**Figure 7:**
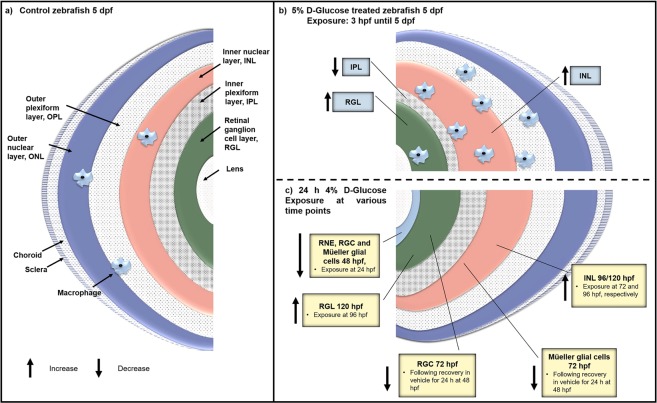
Diagrammatic representation of the impact of hyperglycaemia on retinal patterning during zebrafish embryogenesis. (**a**) Posterior half of control zebrafish eye at 5 dpf representing the normal patterning of the different retinal cell layer sub-types. **(b)** Retinal cell layer patterning following pulsatile 5% D-Glucose exposure from 3 hpf until 5 dpf. RGL and INL exhibit an increase, while IPL exhibits decreased thickness at 5 dpf, and OPL and ONL remain unaffected compared to untreated controls. Increased number of macrophages accumulate in the embryonic retina at 5 dpf following 5% D-Glucose exposure from 3 hpf until 5 dpf as compared to untreated controls. **(c**) Retinal cell layer patterning following a 24 h 4% D-Glucose exposure at various time points. RNE, present in the embryonic eye from 24 until 48 hpf exhibits decreased thickness at 48 hpf following 4% D-Glucose exposure at 24 hpf as compared to untreated controls. RGL, unaffected at 96 hpf following exposure at 72 hpf, exhibits increased thickness at 120 hpf following exposure at 96 hpf compared to untreated controls. The IPL exhibited no change in thickness at 96 and 120 hpf following exposure at 72 and 96 hpf, respectively. INL shows increased thickness at 96 and 120 hpf following exposure at 72 and 96 hpf, respectively, and OPL and ONL remain unaffected at 96 and 120 hpf following exposure at 72 and 96 hpf, respectively compared to untreated controls. RGC and Müeller glial cell number at 48 hpf declines following exposure at 24 hpf as compared to untreated controls. The RGC and Müeller glial cell number fails to recover at 72 hpf following a 24 h recovery period in the vehicle starting at 48 hpf as compared to untreated controls. RNE, retinal neural epithelium; RGL, retinal ganglion layer; IPL, inner plexiform layer; INL, inner nuclear layer; OPL, outer plexiform layer; ONL, outer nuclear layer; RGC, retinal ganglion cell.

The zebrafish RNE is the site for neurogenesis, and neuroepithelial cells within the RNE are crucial for morphogenesis, cell motility and axonal guidance during early retinal development^46,47^. Neuroepithelial cells exit the cell cycle during the course of neurogenesis and differentiate into RGCs and Müeller glial cells^48^. Correct development of the RNE is therefore crucial for the precise stratified appearance of various retinal cell layers within the wild type retina (Fig. 7a). The decrease in the number of RGCs and Müeller glial cells following glucose exposure (24–48 hpf), appears be a direct consequence of RNE thinning (Fig. 7c). RNE is a primordial tissue that gives rise to various cell types within the retina^26^. Therefore, early disruption of the RNE due to hyperglycemia may have dramatic consequences in retinal organization in the embryos and later during adulthood (Fig. 7b,c).

Following high-glucose exposure, zebrafish embryos exhibited altered thickness of various retinal layers at 5 dpf (Fig. 7b). We found RGL thickening at 5 dpf in the zebrafish embryos exposed to 5% D-Glucose from 3 hpf until 5 dpf. Thickening of the RGL is a consequence of increased intraocular inflammation^49^. Intraocular inflammation results from the disruption of the blood-aqueous barrier and increased protein concentrations in the aqueous humour in patients with diabetes^50^. This results in a concomitant loss of photoreceptors causing progression of visual disorders^49^.

We noted a significant decrease in the thickness of the IPL at the same time in the zebrafish embryos (Fig. 7b). The IPL consists of synapses formed by the dendrites of the ganglion cells and the axons of the bipolar cells. Reduced IPL thickness can have severe downstream effects on vision by initiating the atrophy of neuronal processes within the retina^51,52^. This suggests that hyperglycaemia during zebrafish retinal development can influence vision loss because of reduced IPL thickness. The reduced IPL thickness is consistent with previous results from streptozotocin (STZ) induced diabetes in rats^51^. In another study, a 22% decrease in IPL thickness was observed 7.5 months following STZ induced diabetes in rats, suggesting a cumulative loss of neuronal dendrites and synapses in the retina^53^.

Thickening of the INL is a feature in our model of retina development in zebrafish under high-glucose exposure (Fig. 7b,c). Increased INL thickness represents activation and hypertrophy of the Muller glial cells^54^. Muller glial cells mediate the key relationships such as metabolism, ion homeostasis and phagocytosis of neuronal debris, between the retinal vessels and neurons and thus, their activation because of INL thickening may induce progressive neuronal loss, contributing to vision loss^55–58^. Additionally, the reduced presence of BrdU labelled cells in the embryonic zebrafish retina following high-glucose exposure during development suggests a decrease in the retinal progenitor cells as well as the differentiated cells, leading to the loss of various retinal cell populations. Decreased number of BrdU labelled cells in a rat model of diabetes has been linked to early diabetic retinal disease leading to increased VEGF and fibronectin expression, and greater blood retinal barrier breakdown^59^.

We did not detect a change in the thickness of the OPL or ONL following high-glucose exposure (Fig. 7b,c) suggesting that the outer retina is not susceptible to hyperglycaemia during retina formation.

Emerging evidence now suggests that prior to clinically detectable vision disorders retinal neurons and Müeller glial cells undergo a change in function (reviewed in Fletcher *et al*., 2007), which is evidence supporting the notion that RGCs and Müeller glial cells play important roles in maintaining healthy vision. RGCs for example, transfer visual information in the form of action potential to various regions of the brain^60^. Similarly, retinal Müeller glial cells play an indirect role in maintaining a healthy retina by the maintaining the integrity of the blood-retinal barrier^61^. Müeller glial cell dysfunction and death have been suggested to cause neuronal and vascular injury through loss of the neuroprotective mechanisms like secretion of trophic factors and restoration of imbalanced neurotransmitter, ion and water concentrations to normal physiological levels by Müeller glial cells^62^. Changes in the number and function of retinal neurons and Müeller glial cells have been proposed to play a role in exacerbating visual disorders in diabetes^61^.

Our results show not only a significant and severe reduction in the RGCs and Müeller glial cell number during the periods of glucose exposure between 24 and 48 hpf but also after a 24 h period of recovery when imaged at 72 hpf. This indicates that the major visual and non-visual functions supported by these cells in the embryonic retina are compromised under maternal hyperglycaemic conditions. Disorders acquired during foetal organ development as a result of maternal malnutrition are considered irrecoverable during the post-natal life because of foetal programming^63^. This raises serious concerns about visual outcomes because of poor retinal development in babies born to women suffering from pre-existing diabetes or gestational diabetes.

A variety of microvascular changes like increased retinal blood flow^64^, vascular occlusion^65^, haemorrhages^66^, micro-aneurysms^67^ and retinal blood barrier breakdown^68^ have been reported in individuals with diabetes. The increased staining for haemoglobin in the embryonic zebrafish retina following high-glucose exposure may be indicative of the microvascular damage.

Persistent hyperglycaemia initiates chronic inflammation of retinal capillary wall inflammation and matrix metalloproteinases induced remodelling of the capillary basement membrane^69^. This culminates in retinal blood vessel destruction and increased leakage of macrophages^69^. Such a phenomenon is a result of increased levels of vascular endothelial growth factor (VEGF), IL-1β, TNF-α, caspase 3 and glutamate in the retinal blood vessels^69^. TNF-α, in turn, leads to increased production of the intercellular adhesion molecule 1 (ICAM-1) and vascular cell adhesion molecule (VCAM) leading to increased leakage of macrophages from the retinal capillaries^69^. This in turn also exacerbates the loss of RGCs as a result of neurotoxicity that ultimately damages the retina leading to vision loss^69^. Therefore, the increased accumulation of macrophages in the embryonic zebrafish retina may be a result of leakage from the choroid and hyaloid retinal blood vessels. This also suggests that the RGC loss in the zebrafish retina may also be a result of hyperglycaemia induced neurotoxicity in the retina.

Despite the absence of *in-utero* exposure of glucose in zebrafish embryos, simulation of gestational hyperglycaemia by direct glucose exposure in our model induced numerous defects in the embryonic retina. We hypothesise that increased hyaloid retinal blood vessel sprouts in the adult zebrafish retina maybe a case of foetal programming due to detrimental changes acquired by the retina during embryogenesis.

Gestational hyperglycaemia increases the risk for developing neurodevelopmental problems, increased risk of obesity, impaired glucose tolerance, impaired insulin secretion, hypertension and cardiovascular complications in post-natal life^11–13,70,71^. Here, we show a previously unrecognised effect of maternal hyperglycaemia on the eye with effects similar to those reported in cases of retinopathy. This suggests merit in early screening for visual disorders in children born to mothers with either gestational or pre-existing diabetes.

In summary, our data demonstrates a previously unrecognised effect of hyperglycaemia on the developing retina that persists into adulthood. Our data indicates that early and regular ophthalmological follow-up may be beneficial in infants born to mothers with diabetes as a means to prevent or mitigate visual loss.

## Methods

### Zebrafish maintenance

All animal husbandry and experimental protocols were approved by the Deakin University animal ethics committee (Approval number: AWC G17–2015). Zebrafish were treated in accordance with the guidelines established by the Deakin University animal ethics committee. Embryos were obtained from adult wild-type (WT), *Tg (fli1:EGFP)*, *Tg (shh:GFP)*, *Tg (gfap:GFP)*, and *Tg (mpeg:DsRed)* and raised in embryonic media (E3) under standard conditions at 28.5 °C with a 14 h light/10 h dark cycle, and staged according to standard procedures^72^.

### High glucose exposure and measurement of total free glucose levels

100 wild-type (WT) zebrafish embryos were placed in 30 ml vehicle in petri plates (Embryonic media, E3) with various concentration of D-Glucose (4% and 5% w/v) at 28.5 °C at 3 hours post fertilisation (hpf). Control embryos were maintained under identical conditions in vehicle only. The environment of the embryos was alternated every 24 h between the various glucose concentrations and vehicle alone until 5 days post fertilisation (dpf) to induce hyperglycaemia in a fluctuating manner using a methodology modified from Gleeson *et al*.^52^ (see Fig. 1a for treatment protocol). For the sake of simplicity, we refer to this exposure as a ‘pulsatile high-glucose exposure’ throughout the text. For humane killing at 5 dpf, embryos were transferred to a 50 ml facon tube and immersed in a bath of iced water. Following confirmation of the absence of heart beat under a using a stereo microscope (Nikon SMZ 745) equipped with a 10X objective, embryos were rinsed thrice in the vehicle. The vehicle was completely aspirated and embryos were homogenised using a hand homogeniser, and centrifuged at 14,000 rev/min for 2 min. 1.5 μl of the supernatant was placed on a glucometer strip (Accu-Chek Performa). Total free glucose levels were measured using a glucometer (Accu-Chek Performa Nano). 3 measurements were taken per sample from 3 biological replicates (n = 3). mean ± s.e.m. for each sample was calculated. Student’s t-test (two-tailed) was performed to analyse statistical significance.

### Histology and hematoxylin and eosin staining

Embryos fixed in 4% paraformaldehyde (PFA) were dehydrated through several baths of absolute ethanol, then in butanol and finally embedded in paraplast for 3 µm transverse sections. Hematoxylin and eosin staining was performed according to standard protocol^73^. Sections were mounted in eukitt and imaged using a bright field microscope (Zeiss Axio Imager A2) with a 20X objective. The thickness of each retinal cell layer was measured by drawing a line across the thickness of each layer using Fiji (software), followed by measuring the length of the line in microns (µm). Average of 3 measurements was recorded by measuring thickness at 3 different positions using the Fiji software for each layer to account for the variability in thickness of the retinal layers at different positions within the same section. 10 sections from 5 biological replicates (n = 5) were analysed to quantify the retinal layer thickness. Embryos were also imaged using a bright field microscope (Zeiss Axio Imager A2) with a 20X objective. Phenotypic assessment of the size of the eye was carried out by measuring the maximum and minimum diameter of the eye using Fiji. Aspect ratio defined as ‘the ratio of the maximum and minimum diameter’ was subsequently calculated^74^. Aspect ratio provides a measure of the circularity of an object for example; a circle has an aspect ratio of 1:1 and higher values indicate that the shape is elliptical or fusiform^74^. Therefore, we measured aspect ratio as mean ± s.e.m. for each sample (whole-mounted embryos) from 14 biological replicates (n = 14), to provide an assessment of the phenotype of the eye following glucose exposure. Student’s t-test (two-tailed) was performed to analyse statistical significance.

### Analysis of retinal cell proliferation

WT embryos subjected to pulsatile 4 and 5% D-Glucose exposure from 3 hpf until 5 dpf were treated with 10 mM Bromodeoxyuridine (BrdU) to label proliferating cells according to well established methodlogy^75^. Briefly, emrbyos were incubated in 10 mM BrdU for 20 min on ice to allow the uptake of BrdU, followed by incubation in vehicle for exactly 5 min, and fixation in 4% PFA for 2 h at room temperature. Embryos were transferred to 100% Methanol and kept at −20 °C until further use. Embryos were gradually rehydrated in graded washes of methanol:PBTween (3:1, 1:1, 1:3) for 5 min each, followed by 2 washes in 1X PBTween for 5 min each, and fixing in 4% PFA for exactly 20 min. Following fixation, embryos were wahsed thrice in 1X PBTween, and followed by 2 washes with 4 M HCl for 1 minute each. Embryos were then incubated for exactly 20 minutes in 4 M HCl, followed by 5 washes for 10 min each in 1X PBTween. The embryos were then blocked for 30 min using 0.5% Blocking solution and incubated in monoclonal mouse anti-BrdU antibody at a dilution of 1:100 in blocking solution and incubated overnight at 4 °C. Embryos were rnsed for 5 times in 1X PBTween for 5 min each, and incuabated for 2 h at room temperature with horseradish peroxidase conjugated anti mouse secondary antibody (1:100 dilution in 0.1% PBTween). Following 5 washes in PBTween for 10 min each, embryos were incubated in BCL solution and stained using 4-Nitro blue tetrazolium chloride and 5-Bromo-4-Chloro-3-Indoyl Phosphate solution^75^. Following staining, embryos were mounted in 70% glycerol/PBTween and imaged with a 20X objective using a bright field microscope (Zeiss Axio Imager A2). The focus was adjusted in real-time to capture the maximum number of BrdU labelled cells at varying depths of the retina. BrdU labelled cells in the retina were quantified from 5 biological replicates (n = 5) manually throughout the retina using the mutli-point tool in Fiji. mean ± s.e.m. for each sample was calculated and student’s t-test (two-tailed) was performed to analyse statistical significance.

### Analysis of retinal cell population

Various transgenic zebrafish lines were used to study the changes in retinal cell populations, vasculature and blood vessel leakage using confocal microscopy (refer Table 1).

**Table 1:** Zebrafish transgenic lines used in the study.

Transgenic line	Transgene	Phenotype
*Tg* (*fli1*:egfp)	Friend leukaemia integration 1 transcription factor	Green fluorescent blood vessels
*Tg* (*shh*:gfp)	Sonic Hedgehog signalling molecule	Green fluorescent retinal ganglion cells
*Tg* (*gfap*:GFP)	Glial fibrillary acidic protein	Green fluorescent Müeller glial cells
*Tg* (*mpeg*:dsRed)	Macrophage expressed gene	Red fluorescent macrophages
*Tg* (*fli1*:egfp) X *Tg* (*mpeg*:dsRed)	Friend leukaemia integration 1 transcription factor and Macrophage expressed gene	Green fluorescent blood vessels and Red fluorescent macrophages

*Tg (shh:GFP)* embryos were used to visualise and quantify the retinal ganglion cells (RGCs) as the Sonic Hedgehog signalling (*shh*) molecule promotor in the *Tg (shh:GFP)* embryos drives the expression of green fluorescent protein (GFP) in the RGCs.

Similarly, *Tg (gfap:GFP)* embryos were used for the quantification of Müeller glial cells in the retina. The glial fibrillary acidic protein (*gfap*) promotor drives the expression of GFP in Müeller glial cells thereby permitting the visualisation of Müeller glial cells in the retina.

*Tg (shh:GFP)* and *Tg (gfap:GFP)* embryos were exposed to vehicle and 4% D-Glucose from 24 to 48 hpf, then transferred for recovery in the vehicle for 24 h until 72 hpf. Control embryos remained in vehicle alone under identical conditions. 4% D-Glucose treated embryos and controls (n = 10) were anaesthetized using tricaine, mounted on agarose-coated plates using low-melt agarose (LMA) and imaged at 48 hpf and at 72 hpf in a Leica SP 8 confocal microscope using a 20 × 1.0 N/A water immersion objective. Z-stacks were merged to form a 3D image and fluorescent cells counted using the 3D Objects Counter tool (Fiji). mean ± s.e.m. for each sample was calculated from 10 biological replicates (n = 10). Student’s t-test (two-tailed) was performed to analyse statistical significance.

### Analysis of retinal vasculature and microvascular changes

WT embryos exposed to 4 and 5% D-Glucose from 3 hpf until 5 dpf in a pulsatile manner were stained with O-dianisidine solution for the detection of haemoglobin. Briefly, control and glucose-exposed embryos were euthanized at 5 dpf using tricaine. 1.5 ml of O-dianisidine working solution (40% ethanol with 0.01 M sodium acetate, 0.65% H_2_O_2_, and 0.6 mg/mL O*-*dianisidine) was added to each group. Following incubation in dark (15 min), embryos were rinsed twice with PBTween (0.1% Tween-20 in 1X PBS) and fixed overnight using 4% PFA at 4 °C. Stained embryos were incubated in 30 and 50% glycerol/PBTween (10 min each), and stored in 70% glycerol/PBTween. Embryos were mounted in 70% glycerol/PBTween and imaged using bright field microscopy.

We also used *Tg (fli1:EGFP)* zebrafish line where the friend leukaemia integration 1 (*fli11*) transcription factor promotor drives the expression of the enhanced GFP (EGFP) in all blood vessels to visualise changes to hyaloid and choroid retinal blood vessels. In addition, we used *Tg (mpeg:DsRed)* zebrafish line, where the macrophage expressed gene (*mpeg*) promotor drives the expression of Discosoma sp. red (*DsRed*) fluorescent protein in macrophages to visualise the leakage of macrophages from retinal blood vessels.

In order to aid in the simultaneous visualisation of retinal blood vessels (Choroid and hyaloid) and leakage, we obtained double transgenic embryos from a cross of *Tg (fli1:EGFP)* and *Tg (mpeg:DsRed)* adult zebrafish. *Tg (fli1:EGFP)* X *Tg (mpeg:DsRed)* embryos were exposed to 4 and 5% D-Glucose from 3 hpf until 5 dpf according to the established pulsatile glucose exposure. Control embryos remained in vehicle from 3 hpf until 5 dpf. Control, 4 and 5% D-Glucose exposed embryos (n = 10) were anaesthetized using tricaine and mounted on agarose-coated plates using LMA and imaged in a Leica SP 8 confocal microscope using a 20 × 1.0 N/A water immersion objective to study retinal vasculature and blood vessel leakage at 5 dpf using confocal microscopy. mean ± s.e.m. for each sample was calculated from 10 biological replicates (n = 10). Student’s t-test (two-tailed) was performed to analyse statistical significance.

### Retina flat mount and analysis of retinal vasculature

*Tg (fli1:EGFP)* embryos were exposed to vehicle, 4 and 5% D-Glucose from 3 hpf until 5 dpf using the pulsatile exposure method. Embryos were fed thrice daily with *Paramecium caudatum* until they were capable of eating *Artemia*. Otohime A was used as the initial dry feed, which was further graduated to Otohime B1, and finally onto Otohime S1 as the gape size increased. Adult zebrafish (n = 9) were maintained for each experimental group in 1 litre tanks. Adult zebrafish were humanely killed at 100 dpf. Eyes were enucleated and fixed with 4% PFA at 4 °C for 24 h. Fixed eyes were then transferred to 1X PBTween. Retinas were dissected according to established methodology^76^. Retinas were incubated in 30 and 50% glycerol/PBTween (10 min each), and stored in 70% glycerol/PBTween. Retinas were mounted in 70% glycerol/PBTween on a glass slide and imaged using confocal microscopy (Olympus Fluoview FV10i), and the number of hyaloid retinal blood vessel sprouts were counted. Significance of the results was calculated using Student’s t-test (two-tailed). mean ± s.e.m. for each sample was calculated from 5 biological replicates (n = 5). Student’s t-test (two-tailed) was performed to analyse statistical significance.

## Supplementary information

S1

S1

## Data Availability

The datasets generated during and/or analysed during the current study are not publicly available due to being part of an unsubmitted thesis but are available from the corresponding author on reasonable request.
