# Supplementary figures and images for: High glucose levels affect retinal patterning during zebrafish embryogenesis
Source: Sci Rep. 2019 Mar 11;9:4121. doi: 10.1038/s41598-019-41009-3 (PMC6411978; doi:10.1038/s41598-019-41009-3)

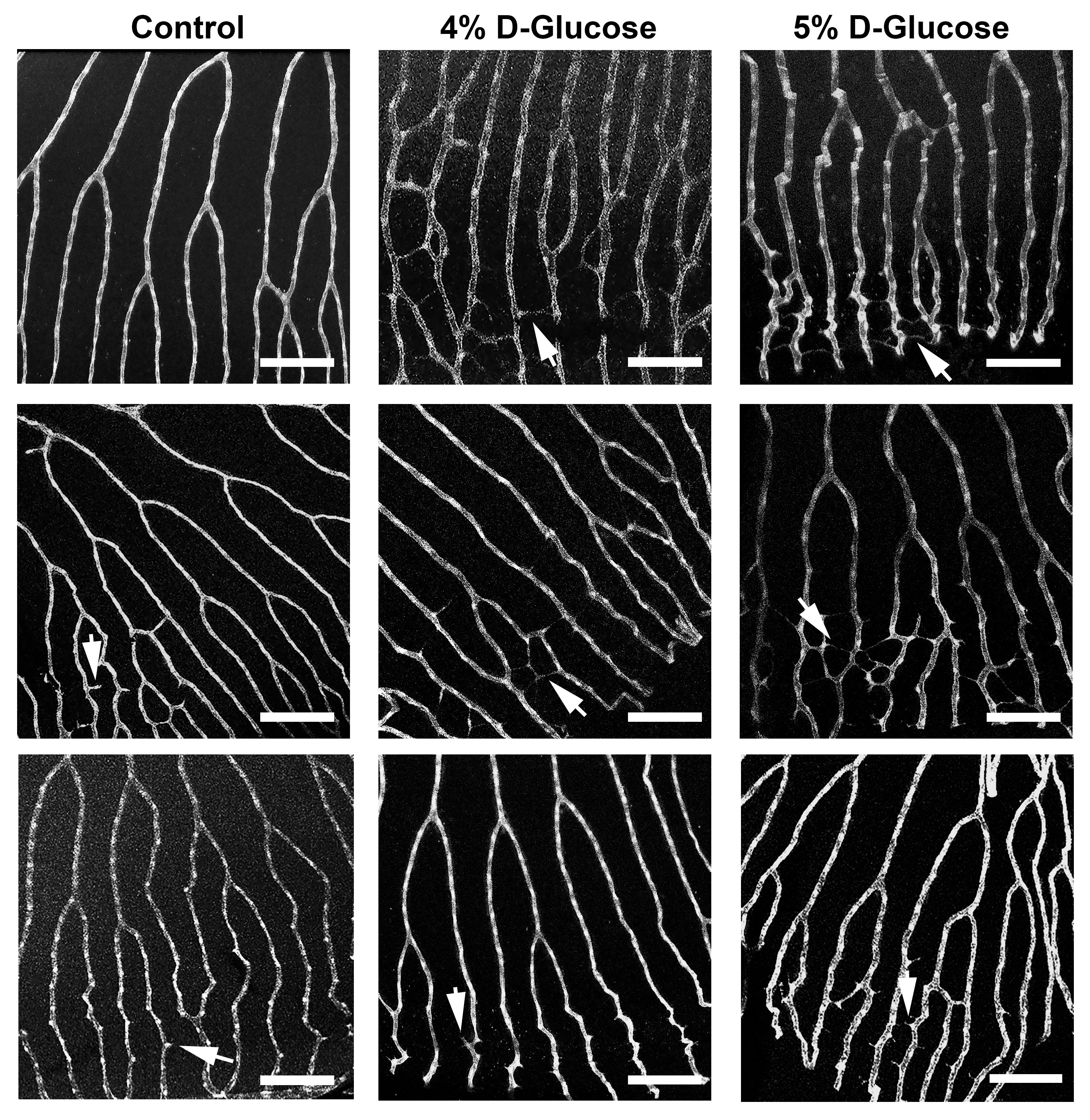

Supplement: Supplementary file 1 — S1 [file 41598_2019_41009_MOESM1_ESM.tif]
