# Supplementary material for: High glucose levels affect retinal patterning during zebrafish embryogenesis
Source: Sci Rep. 2019 Mar 11;9:4121. doi: 10.1038/s41598-019-41009-3 (PMC6411978; doi:10.1038/s41598-019-41009-3)
Supplement: Supplementary file 2 — S1 [file 41598_2019_41009_MOESM2_ESM.docx]

# **Supplementary Data**

**Supplementary Figure S1. Long-term implications of high-glucose exposure during embryonic stages in the adult zebrafish retina.** Retinal vasculature of *Tg (fli1:EGFP)* zebrafish adults (100 dpf) exposed to vehicle, 4 and 5% D-Glucose from 3 hpf until 5 dpf in a pulsatile manner. 4 and 5 % D-Glucose treated group exhibited an increase in the number of hyaloid blood vessel sprouts (indicated by white arrows). Scale bar, 100 µm. Images inverted to grayscale.
